# A case report of *Talaromyces marneffei* Oro-pharyngo-laryngitis: a rare manifestation of Talaromycosis

**DOI:** 10.1186/s12879-019-4650-7

**Published:** 2019-12-05

**Authors:** Thanyarak Wongkamhla, Piriyaporn Chongtrakool, Anupop Jitmuang

**Affiliations:** 1 Division of Infectious Diseases and Tropical Medicine, Department of Medicine, Faculty of Medicine Siriraj Hospital, Mahidol University, 2 Wanglang Road, Bangkoknoi, Bangkok, 10700 Thailand; 2 Department of Microbiology, Faculty of Medicine Siriraj Hospital, Mahidol University, Bangkok, Thailand

**Keywords:** *Taralomyces marneffei*, Talaromycosis, Oro-pharyngo-laryngitis, Anti-interferon-gamma autoantibodies

## Abstract

**Background:**

The incidence of *Taralomyces marneffei* infection in HIV-infected individuals has been decreasing, whereas its rate is rising among non-HIV immunodeficient persons, particularly patients with anti-interferon-gamma autoantibodies*. T. marneffei* usually causes invasive and disseminated infections, including fungemia. *T. marneffei* oro-pharyngo-laryngitis is an unusual manifestation of talaromycosis.

**Case presentation:**

A 52-year-old Thai woman had been diagnosed anti-IFNɣ autoantibodies for 4 years. She had a sore throat, odynophagia, and hoarseness for 3 weeks. She also had febrile symptoms and lost 5 kg in weight. Physical examination revealed marked swelling and hyperemia of both sides of the tonsils, the uvula and palatal arches including a swelling of the epiglottis, and arytenoid. The right tonsillar biopsy exhibited a few intracellular oval and elongated yeast-like organisms with some central transverse septum seen, which subsequently grew a few colonies of *T. marneffei* on fungal cultures. The patient received amphotericin B deoxycholate 45 mg/dayfor 1 weeks, followed by oral itraconazole 400 mg/day for several months. Her symptoms completely resolved without complication.

**Conclusion:**

In patients with anti-IFN-ɣ autoantibodies, *T. marneffei* can rarely cause a local infection involving oropharynx and larynx. Fungal culture and pathological examination are warranted for diagnosis *T*. *marneffei* oro-pharyngo-laryngitis. This condition requires a long term antifungal therapy.

## Background

*Talaromyces marneffei* (formerly known as *Penicillium marneffei*) is an important dimorphic fungus; it is the only member in the genus causing systemic mycosis and is more prevalent in Southeast Asia [1–3]. Historically, human *T. marneffei* infection has been confined to patients with acquired immunodeficiency syndrome (AIDS) [1]. In recent years, the incidence of *T. marneffei* infection in those populations has been decreasing following treatment with highly active antiretroviral regimens and preventive measurements. However, the rate of this infection in non-HIV-infected individuals has been rising, particularly in patients with anti-interferon-gamma autoantibodies (anti-IFNɣ autoantibodies), patients receiving systemic corticosteroids or immunosuppressive agents, organ transplant recipients, and patients receiving novel anti-cancer targeted therapies [4]. When *T. marneffei* infects those populations, it usually causes fungemia and disseminated disease to various organs, such as the skin, lymph node, lung, spleen, and bone [4, 5].

Oro-pharyngo-laryngitis caused by *T. marneffei* is a very uncommon talaromycosis, and has been rarely reported, with previous case reports of *T. marneffei* oro-pharyngitis and pharyngo-laryngitis limited to patients with underlying AIDS [6–8]. Herein, we report a rare manifestation of talaromycosis in a woman who had underlying anti-IFNɣ autoantibodies. She presented with subacute oro-pharyngo-laryngitis, which was rapidly resolved following a systemic antifungal therapy.

(This work was presented in part at the 9th Trends in Medical Mycology (TIMM) Meeting, October 11–14, 2019 in Nice, France)

### Case presentation

**Fig. 1:**
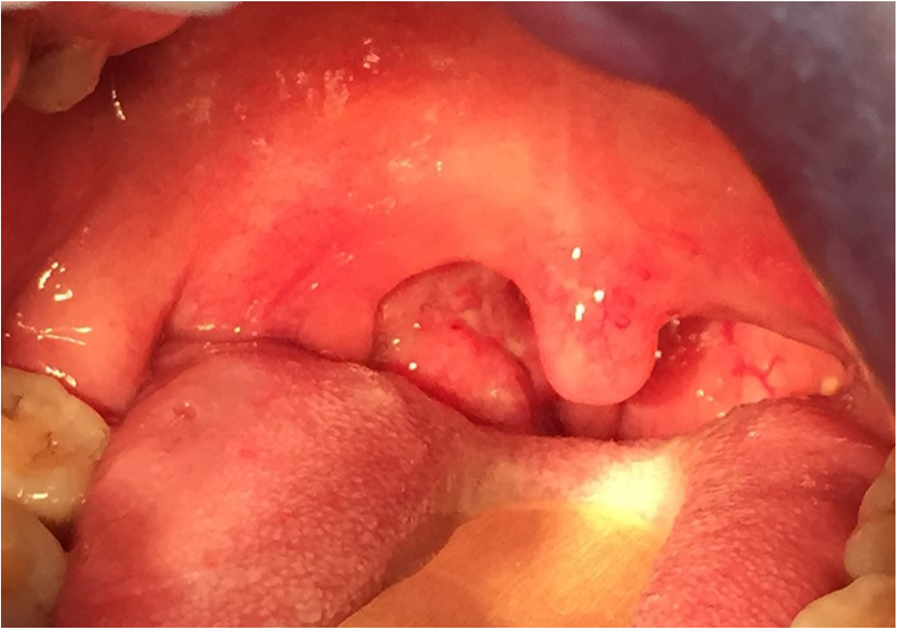
Physical examination revealed marked swelling and hyperemia of both sides of the tonsils, including the uvula and palatal arches

**Fig. 2:**
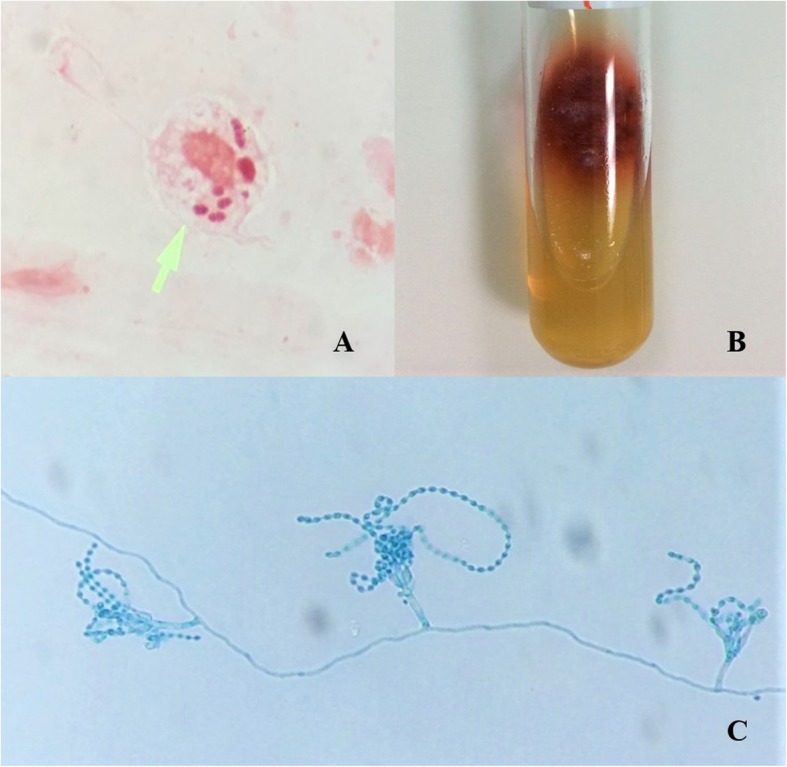
**a** Tissue Gram stain from the right tonsillar biopsy exhibited a few intracellular oval and elongated yeast-like organisms with some central transverse septum seen, **b** they subsequently grew a few mold colonies with the typical diffusible red-colored pigment on fungal cultures, **c** the lactophenol cotton blue microscopic examination demonstrated hyaline septate molds, with branched and non-branched conidiophores consisting of brush-like phialides with long chains of round and elliptical conidia

A 52-year-old Thai woman had been diagnosed anti-IFNɣ autoantibodies for 4 years. Four years ago (March 2015), she presented with prolonged fever, a weight loss of approximately 10 kg, bilateral tonsillar enlargement, and multiple cervical lymphadenopathy. A lymph node biopsy from the left cervical node showed the growth of *Mycobacterium absessus*. Disseminated *Mycobacterium abscessus* infection was diagnosed. She denied using illicit drugs, herbal medicines, or corticosteroids. Immunological studies, including anti-HIV testing, were all negative, but anti-IFNɣ autoantibodies tested highly positive. She received intravenous imipenem and amikacin for a primary anti-mycobacterial therapy, which were later switched to oral clarithromycin and ciprofloxacin for maintenance therapy. She had relapse infections twice during the course of treatment. Thereafter, the anti-mycobacterial regimen was changed to oral clarithromycin and linezolid. Following the new regimen, she complied well with the treatment, and her condition was in remission for 1 year. Before this admission, she had a sore throat, which was particularly more painful at the right side of the pharynx, odynophagia, and hoarseness for 3 weeks. She also had febrile symptoms and lost 5 kg in weight. She received oral amoxicillin 1.5 g/day from a primary physician, but her symptoms did not resolve. She denied foreign body sensation, and had no dysphagia, stridor, or difficult breathing. Physical examination revealed marked swelling and hyperemia of both sides of the tonsils, including the uvula and palatal arches (Fig. 1), and a single left submandibular lymph node, sized approximately 1 cm, was identified. Indirect laryngoscopy demonstrated a moderate swelling of the epiglottis, arytenoid, and vocal cord with normal airway opening. There were no skin papules or nodules, including no hepatosplenomegaly found. Blood chemistries, including plain chest radiography, were unremarkable. The patient was performed right tonsillar biopsy at 1 day following admission. The tissue biopsy Gram stain (Fig. 2a), including the pathological sections, exhibited a few intracellular oval and elongated yeast-like organisms with some central transverse septum seen with dense small lymphoid cell and plasma cell infiltrates. Acid fast and modified acid fast staining from the pathological sections were negative. One week later, the tissue biopsy grew few mold colonies with the typical diffusible red-colored pigment on the fungal cultures (Fig. 2b). Morphological identification based on lactophenol cotton blue microscopic examination demonstrated these to be hyaline septate molds, with branched and non-branched conidiophores consisting of brush-like phialides with long chains of round and elliptical conidia (Fig. 2c). All of the findings were suggestive of *T. marneffei*. However, antifungal susceptibility testing was not performed due to there being no standard breakpoint criteria for interpretation. The tissue sent for bacterial and mycobacterial culture showed no significant growth. Routine blood cultures (BD BACTEC™, Becton, Dickinson and Company), including fungal and mycobacterial blood cultures (BD BACTEC™ Myco/F Lytic, Becton, Dickinson and Company) were negative for any microorganisms. Thus, no evidence of relapsed mycobacterial disease or other pathogen co-infections was detected.

Following the diagnosis of *T. marneffei* infection, amphotericin B deoxycholate 45 mg/day (0.6 mg/kg/day) was commenced as the primary antifungal therapy. The patient continued receiving oral clarithromycin and linezolid for *M. abscessus* maintenance therapy. Following 5 days of amphotericin therapy, the patient developed acute kidney injury with a rising creatinine level from 0.86. to 2.44 mg/dL. Her blood potassium, magnesium, and bicarbonate levels were 3.4, 1.7, and 24 mEq/L, respectively. Finally, the antifungal regimen was changed to itraconazole oral solution with a daily dose of 400 mg/day after receiving amphotericin B for 5 days. The presenting symptoms of a sore throat and odynophagia disappeared after 1 week of the antifungal therapy. Four weeks after the treatment, the swelling of the pharynx and larynx were markedly reduced. She was continued with itraconazole for 4 months, and later a dose of 200 mg/day was used for long-term secondary prophylaxis. Her symptoms completely resolved with no complication. She still has been receiving the itraconazole prophylaxis until now.

## Discussion

*T. marneffei* infection is an opportunistic fungal infection that is increasingly reported among non-HIV infected adults and children [9–13]. A previous cohort study from Thailand reported that 22 from 34 non-HIV infected cases with *T. marneffei* infections had no previously known underlying diseases and no apparent immunodeficiencies [11]. Of those 22 cases, blood samples from 9 cases were tested for immunological studies, and all were positive for anti-IFNɣ autoantibodies by ELISA. According to the study, the anti-IFNɣ autoantibody may be a newly associated risk factor for *T. marneffei* infection in non-HIV infected individuals [11]. Anti-IFNɣ autoantibody is an acquired adult onset immunodeficient condition and its pathophysiology has not yet been clearly described. We still do not know how the autoantibodies are acquired, where from, and why this immunodeficient condition is usually found in adulthood, particularly in Asian populations. Some specific HLA classes, such as HLA-DRB1, and HLA-DQB1 commonly discovered in Asian populations were proposed to be associated with this condition [14, 15]. Clinical infections of patients with anti-IFNɣ autoantibodies are usually disseminated diseases caused by opportunistic pathogens, such as non-tuberculous mycobacteria, particularly rapidly growing mycobacteria, *Salmonella* non-Typhi, *Cryptococcus*, *Histoplasma*, *Talaromyces*, and the Varicella-Zoster virus [16, 17]. Similar to this case report, more than half of patients developed relapsed infection from a prior infected organism or recurrent infection from a new opportunistic organism despite receiving long-term antimicrobial treatment [17].

*T. marneffei* infection in both HIV-infected and non-HIV infected individuals usually manifests as fungemia and disseminated disease to various organs [11, 13, 18]. This case report presented with a subacute onset of oro-pharyngo-laryngitis caused by *T. marneffei*. *T. marneffei* oro-pharyngo-laryngitis is a rare and atypical presentation of talaromycosis. Previous case reports, including the present case, are summarized in Table 1 [6–8, 19–21]. Most of the case reports, including the present case, had underlying immunodeficiencies as predisposing factors. In HIV-infected patients with low CD_4_ T cell counts, *T. marneffei* oro-pharyngo-laryngitis may present as a part of disseminated talaromycosis. Several case reports demonstrated that oro-pharyngo-laryngitis coincided with systemic involvements, such as multiple skin lesions, lymphadenopthy, and hepatosplenomegaly [6, 9, 21]. This infection presented as subacute or chronic painful papular or ulcerative lesions localized to the oropharyngeal area or progressed to involve the laryngeal area, and sometimes it would not be recognized by the clinician. Presenting symptoms vary based on the area of mucosal involvement, such as painful oropharyngeal lesions, odynophagia, and hoarseness. However, painful oropharyngeal lesions in HIV-infected individuals have several etiologies, such as aphthous ulceration, herpes simplex infection, erythema multiforme, lymphoma, and histoplasmosis [19, 22]. Thus, the diagnosis of *T. marneffei* oro-pharyngo-laryngitis requires tissue microscopic examination and a histopathological finding of spherical, oval, and elongated yeast cells with clear central transverse septations, according to the case reports in Table 1. However, the morphological identification of *T. marneffei* from a tissue section is not specific because its morphology is occasionally identical to *Histoplasma capsulatum* and some *Candida* spp., so tissue fungal culture and isolation are warranted for confirmed diagnosis. The present case was undergoing tonsillar biopsy for microbiological diagnosis very early because recurrent mycobacterial infection and invasive mycoses were highly suspected. In this case, microscopic examination with tonsillar biopsy Gram stain could provide a presumptive diagnosis of talaromycosis (Fig. 2a), and later the tissue fungal culture showed characteristic colonies of *T. marneffei* (Fig. 2b). A previous study demonstrated an early microscopic examination of the sample could give a presumptive diagnosis before the fungal isolation in approximately 60% of cases [23]. In cases of disseminated infection listed in Table 1, *T. marneffei* were also isolated from blood and urine cultures [6, 7]. The present case was probably not the disseminated infection because the clinical findings showed no evidence of distant organ involvement, including blood culture negative for the fungal isolate. However, the route of *T. marneffei* oro-pharyngo-laryngitis remains unclear, though inhalation or ingestion of contaminated fungal spores may likely be the route of infection. The oropharynx and tonsil are the primary sites for fungal inoculation following inhalation and ingestion. Partial immunity and more inflammatory response to the fungal organism could explain the shorter onset of the patient’s symptoms compared to several reported cases.

**Table 1:** A summary of case reports of *Talaromyces marneffei* oro-pharyngo-laryngitis

Country, no. of case (Year)	Age (yrs.)/ Sex	Predisposing conditions	Duration of symptoms	Clinical manifestations	Diagnosis	Treatments	Outcomes
Thailand, 2 (1997) [19]	29/ F, na/ F	HIV infection	na	Painful oral papules and skin papules (1); Oral ulcerations (1)	Tissue culture, histopathology	na	na
Thailand, 1 (2000) [8]	25/ F	HIV infection	3 months	Sore throat, multiple oral papules	Microscopic exam, tissue culture	Oral KET, ART	Cure
Hong Kong, 1 (2001) [7]	63/ M	HIV infection	2 months	Painful multiple oral ulcerations	Tissue, urine and blood cultures, histopathology	IV AMB, then oral ITRA, ART	Cure
China, 1 (2012) [20]	39/ M	None	4 months	Chronic painful granuloma like oral ulceration	Histopathology, molecular assay, electron microscopy	Oral ITRA	Cure
India, 1 (2017) [21]	33/ M	HIV infection	2 months	Fever, hoarseness, odynophagia, multiple skin papules	Tissue culture, histopathology	IV AMB, then oral ITRA, ART	Cure
China, 7 (2017) [6]	Median 34/ M (7)	HIV infection (7)	na	Fever (7), sore throat (7), regional lymphadenopathy (7), mucosal ulceration (6), skin lesions (5), hepatosplenomegaly (5), hoarseness (4), local mass (4)	Tissue culture, blood culture, histopathology	IV AMB, then oral antifungals (FLU, ITRA) (5), ART (7)	Dead (3), improved (4)
The present case	52/F	Anti-IFNɣ autoantibodies	3 weeks	Fever, sore throat, hoarseness, odynophagia	Microscopic exam, tissue culture, histopathology	IV AMB, then oral ITRA	Cure

*ABD* amphotericin B deoxycholate, *Anti-IFNɣ autoantibodies* anti-interferon-gamma autoantibodies, *ART* antiretroviral therapy, *F* female, *FLU* fluconazole, *HIV* human immunodeficiency virus, *ITRA* itraconazole, *IV* intravenous, *KET* ketoconazole, *M* male, *na* not available, *yrs.* years

A treatment regimen of talaromycosis prefers initial induction with a systemic antifungal agent, particularly intravenous amphotericin B for a few weeks, followed by maintenance oral antifungal therapy, particularly itraconazole, and subsequently a continuation of secondary prophylaxis for several weeks [24]. The treatment regimen is generally applied for HIV-infected or immunodeficient persons who have frequently presented with disseminated infection. For *T. marneffei* oro-pharyngo-laryngitis, the treatment regimen has not yet been clearly defined, but it should be based on severity and host immune status. An oral antifungal therapy alone would be appropriate for a localized infection similar to the previous reports [8, 18], whereas an initial intravenous antifungal therapy should be used for severe or extensive *T. marneffei* infection and for an infection occurring in immunodeficient persons. Most of the previous cases, including the present case, did respond well to the antifungal therapy, although 2 cases died from delayed antifungal therapy and 1 died from unresponsive treatment. Treatment outcomes are also based on host immunity. In HIV-infected persons, antiretroviral treatment should be started early to raise the CD_4_ T cell counts when diagnosing *T. marneffei* infection [6, 18, 24]. Secondary antifungal prophylaxis can be discontinued when the numbers from the CD4 T cell counts are increasing sufficiently regarding the recommendation [24]. However, in patients with anti-IFNɣ autoantibodies, a standard antifungal treatment regimen, including long-term antifungal therapy, has still not been established. The author considered a continuation of long-term antifungal prophylaxis to prevent relapse of the *T. marneffei* infection in this case. In conclusion, in patients with anti-IFN-gamma autoantibodies, *T. marneffei* can rarely cause a local infection involving the pharynx and larynx. Fungal culture, microscopic examination, and histopathological study are warranted for the diagnosis of *T. marneffei* oro-pharyngo-laryngitis. A long-term antifungal therapy was required to successfully treat this infection.

## Data Availability

All data and materials of this article are included in the manuscript and thus available to the reader.

## Abbreviations

ABD: amphotericin B deoxycholate
Anti-IFNɣ autoantibodies: anti-interferon-gamma autoantibodies
ART: antiretroviral therapy
F: female
FLU: fluconazole
HIV: human immunodeficiency virus
ITRA: itraconazole
IV: intravenous
KET: ketoconazole
M: male
na: not available
yrs.: years
